# Erratum to “Bull fertility and semen quality are not correlated with dairy and production traits in Brown Swiss cattle” (JDS Commun. 3:120–125)

**DOI:** 10.3168/jdsc.2022-3-5-378

**Published:** 2022-09-12

**Authors:** Xena Marie Mapel, Maya Hiltpold, Naveen Kumar Kadri, Ulrich Witschi, Hubert Pausch

The animal ethics statement was missing from this paper. The following statement should have been included: **We used animal data that were routinely collected for other purposes, such as genetic evaluation, and ethical approval for the use of animals was thus deemed unnecessary**.

## References

[bib1] Mapel X.M., Hiltpold M., Kadri N.K., Witschi U., Pausch H. (2022). Bull fertility and semen quality are not correlated with dairy and production traits in Brown Swiss cattle. JDS Commun..

